# B_12_ deficiency with neurological manifestations in the absence of anaemia

**DOI:** 10.1186/s13104-015-1437-9

**Published:** 2015-09-18

**Authors:** Dissanayake Mudiyanselage Priyantha Udaya Kumara Ralapanawa, Kushalee Poornima Jayawickreme, Ekanayake Mudiyanselage Madhushanka Ekanayake, Widana Arachchilage Thilak Ananda Jayalath

**Affiliations:** Department of Medicine, University of Peradeniya, Peradeniya, Sri Lanka

**Keywords:** Vitamin B_12_ deficiency, Anaemia, Macrocytosis, Polyneuropathy, Sub-acute combined cord degeneration, Hydroxycobalamine, Sri Lanka

## Abstract

**Background:**

Vitamin B_12_ deficiency is often diagnosed with hematological manifestations of megaloblastic macrocytic anemia, which is usually the initial presentation. Neurological symptoms are often considered to be late manifestations and usually occur after the onset of anemia. Sub acute combined cord degeneration, which is a rare cause of myelopathy is however the commonest neurological manifestation of vitamin B_12_ deficiency.

**Case presentation:**

We present a case of a 66 year old Sinhalese Sri Lankan female, who is a strict vegetarian, presenting with one month’s history suggestive of Sub-acute combined cord degeneration in the absence of haematological manifestations of anaemia. Her Serum B_12_ levels were significantly low, after which she was treated with hydroxycobalamine supplementation, showing marked clinical improvement of symptoms, with normalization of serum B_12_ levels. Hence, the diagnosis of vitamin B_12_ deficiency was confirmed retrospectively.

**Conclusion:**

Vitamin B_12_ deficiency could rarely present with neurological manifestations in the absence of anaemia. Therefore a high index of suspicion is necessary for the early diagnosis and prompt treatment in order to reverse neurological manifestations, as the response to treatment is inversely proportionate to the severity and duration of the disease.

## Background

Vitamin B_12_ deficiency is usually associated with various hematological, gastrointestinal and neuropsychiatric disorders [1]. Hematological manifestations include anemia with a macrocytic blood picture and megaloblastic bone marrow occurring rarely with the involvement of all three cell lines causing pancytopenia. According to studies which have been conducted in the past, anemia or hematological impairment usually starts early and precedes [2] the neuro-psychiatric manifestations which are more serious, and delayed treatment may lead to permanent disability [3].

Neuropsychiatric manifestations include neuropathy, myelopathy, dementia, neuropsychiatric abnormalities and rarely optic atrophy. Sub-acute combined cord degeneration (SACD) is the most frequent manifestation of vitamin B_12_ deficiency [4], but anemia is a common and leading symptom to the diagnosis of vitamin B_12_ deficiency, and neurological manifestations typically occur after the onset of anemia [1, 5].

## Case presentation

A 66 year old Sinhalese Sri Lankan lady, who had been a strict vegetarian for the past 20 years, presented to our teaching hospital with 1 month’s history of numbness and tingling of both lower limbs with unsteadiness of gait, without complaints of urinary or fecal incontinence. She had no fever, night sweats, gastrointestinal symptoms or any uremic symptoms. She did not have a history of pre-existing diabetes mellitus, hypertension or ischemic heart diseases or alcohol consumption. She did not show clinical or biochemical features of pernicious anemia.

On examination, she was a thinly built lady with no hypo or hyperthyroid features. She had no pallor, icterus, peripheral edema or lymphadenopathy. Her pulse rate was 82 beats per minute with a blood pressure of 130/78 mmHg. She did not have hepato-splenomegaly.

On neurological examination, she had stocking type sensory impairment up to mid shin level and absence of joint position and vibration sensation in both lower limbs. Her ankle jerks were absent bilaterally. Romberg’s sign and Babinski sign were positive, but had no impairment of muscle power or tone. Her upper limbs, cranial nerves and higher functions were neurologically normal and had no cerebellar impairment.

Her laboratory investigations were unremarkable, with a hemoglobin concentration of 12.1 g/dl, red blood cell count of 4.39/mm^3^, mean corpuscular volume (MCV) of 83.3 fl, mean corpuscular hemoglobin concentration of 27.6 g/dl, platelet count of 334,000/mm^3^, serum creatinine of 82 mg/dl, and normal fasting plasma glucose level sand thyroid stimulating hormone levels. Her blood picture revealed no features of vitamin B_12_ deficiency and showed normochromic normocytes. Demyelinating polyneuropathy was confirmed with nerve conduction studies.

Even though she did not have features of anemia, the clinical picture was suspicious of vitamin B_12_ deficiency and associated SACD. Hence we proceeded with the serum vitamin B_12_ levels, which was found to be very low; 84.90 pg/ml (208–963). Thus, the clinical diagnosis of sub-acute combined cord degeneration due to deficiency of vitamin B_12_ without anemia was made.

She was treated with intramuscular hydroxycobalamine 1000 μg for 7 days, weekly for 6 weeks and thereafter three monthly. After 3 months of replacement therapy, the patient showed clinical improvement, with repeated B_12_ levels being elevated up to 308.6 pg/ml. Follow up nerve conduction study done at 1 and 3 years showed previously absent Sural sensory nerve action potentials reappearing, and common peroneal and posterior tibial nerve conduction velocities being improved. This case demonstrates early clinical improvement, with slow recovery of polyneuropathy on nerve conduction studies, despite rapid correction of vitamin B_12_ levels following therapy with hydroxycobalamine.

## Discussion

Vitamin B_12_ is a water-soluble vitamin found naturally in meat and food of animal origin. Its physiological functions include erythropoiesis, the synthesis and maintenance of myelin sheath and the synthesis of deoxyribonucleic acid. The current recommended dietary allowance for vitamin B_12_ has been set by the Institute of Medicine at 2.4 μg per day for males and females aged 14 years and above, with a serum level of vitamin B_12_ of 120–180 pmol/l indicative of deficiency [6]. The mechanisms that cause deficiency are malabsorption, malnutrition or genetic deficiency of methylmalonyl-CoA mutase. Based on this case and considering malnutrition as the etiological factor, vegans who are not on vitamin B_12_ supplements or fortified foods containing the vitamin are found to be at a higher risk of developing deficiency, compared to vegetarians [7].

B_12_ deficiency presents with hematological, gastrointestinal and neuropsychiatric manifestations. Neuropsychiatric manifestations commonly associated with deficiency include myelopathy, neuropathy, dementia, neuropsychiatric abnormalities and rarely show optic nerve atrophy. SACD though a rare cause of myelopathy, is the most frequent clinical manifestation of B_12_ deficiency [4]. The clinical signs of SACD typically include a spastic paraparesis, extensor plantar response, and impaired perception of position and vibration, and may have associated peripheral neuropathy which explains this patient’s stocking type numbness and absence of ankle jerks. Neuropathological lesions in SACD have been found to be due to overproduction of the myelinolytic Tumor necrosis factor α (TNFα) and to the reduced synthesis of the two neurotrophic agents; the Epidermal growth factor (EGF) and interleukin-6 caused by vitamin B_12_ deficiency [8]. Neuropathological studies, including T2 weighted Magnetic resonance imaging (MRI), show lesions classically involving the posterior and lateral columns, predominantly in the upper thoracic and midthoracic regions, resulting in concomitant upper limb and lower limb involvement [9], in contrast to this case which has isolated lower limb involvement with normal upper limbs.

Neuropsychiatric manifestations include decreased memory, personality change, psychosis, emotional lability, and rarely delirium or coma, and may be seen in patients without haematological manifestations, or low normal B_12_ levels [5]. However this patient had no such psychiatric manifestations nor anaemia. Unusual neurological manifestations of the disease are cerebellar ataxia, leukoencephalopathy, orthostatic tremors, myoclonus, ophthalmoplegia, catatonia, vocal cord paralysis, a syringomyelia like distribution of motor and sensory deficits, and autonomic dysfunction [10] which were not seen in this case.

Anemia is a common and early symptom leading to the diagnosis of vitamin B_12_ deficiency, while as neurological symptoms are often considered as late manifestations, and are typically preceded by anemia [5], unlike in this rare case of vitamin B_12_ deficiency with neurological manifestations in the absence of anaemia. However, haematological manifestations were found to be associated with neuropsychiatric manifestations in only 28 % in a population study [5]. Approximately two-thirds of patients with vitamin B_12_ deficiency are shown to have hematological abnormalities characterized by anemia, an elevated MCV, the presence of hypersegmented neutrophils and macrocytosis on peripheral blood smears [11]. A previous study has shown 10 % cases to have life threatening haematological manifestations with 5 % having symptomatic pancytopenia [12].

Serum B_12_ level assessment is the mainstay of investigation of vitamin B_12_ deficiency, whereas an increase in serum levels of methylmalonic acid and homocysteine also indicate the diagnosis [13]. Electrophysiological abnormalities include nerve conduction studies suggestive of a sensorimotor axonopathy [14], as seen in this case. MRI of the cervical and dorsal spine shows increased T2 weighted signal intensity in the posterior and lateral columns of the cervical and upper thoracic spinal cord [15].

Once the diagnosis of vitamin B_12_ deficiency has been established, the goals of treatment are to reverse the signs and symptoms of deficiency, replenish body stores and ascertain the cause of deficiency while monitoring the response to therapy. Since the etiology in this case is obviously dietary deficiency, the mainstay of treatment is vitamin B_12_ supplementation. The recommended regimen is 1000 μg intramuscular injections for 5–7 days followed by monthly 500–1000 μg intramuscular injections [16]. Response of the neurologic manifestations is variable and may be incomplete. They usually start around the first week, and is often complete within 6 months [17]. However, response to treatment is inversely proportional to the severity of deficiency, extent of involvement and the time lapsed between onset of symptoms and initiation of therapy [18], indicating the importance of prompt diagnosis and treatment.

## Conclusion

Vitamin B_12_ deficiency though commonly associated with haematological manifestations like macrocytic anaemia, it could rarely present with neurological manifestations in the absence of anaemia or macrocytosis. Therefore a high index of suspicion is necessary in early diagnosis and prompt treatment in order to reverse neurological manifestations, as the response to treatment is inversely proportional to the severity and duration of disease.

## Consent

Written informed consent was obtained from the patient for publication of this case report and accompanying images. A copy of the written consent is available for review by the Editor-in-chief of this journal.

## Abbreviations

SACD: sub-acute combined cord degeneration
MCV: mean corpuscular volume
TNFα: tumor necrosis factor
EGF: epidermal growth factor
MRI: magnetic resonance imaging
